# An intelligence coordination system toward creating the super-intelligent law firm

**DOI:** 10.3389/frai.2023.1145308

**Published:** 2023-07-14

**Authors:** Peter Kaomea

**Affiliations:** Morrison & Foerster, LLP, San Francisco, CA, United States

**Keywords:** super-intelligence, collective-intelligence, pareto, machine intelligence, work coordination, generative artificial intelligence, ChatGPT, work allocation

## Abstract

A large law firm typically exhibits a collective intelligence comprised of hundreds or thousands of legal minds aimed at simultaneously engaging thousands of active matters across scores of industries and dozens of practice specialties with distinct doctrinal and procedural characteristics. The firm is challenged not only to achieve successful, cost-effective outcomes for its clients, but must also simultaneously, in competition with other firms and alternative service providers, attract and cultivate talent, develop and coordinate capabilities across multiple evolving areas of practice and continually improve a robust collective intelligence to gain a competitive edge. As various types of machine intelligences and tools are introduced, firms must also groom these into the collective. In this paper we explore a human-machine hybrid system for addressing this large scale, multi-dimensional, dynamic optimization challenge to coordinate a collective intelligence of humans and machines. Machine intelligence is needed to handle the computational complexity and it is complemented by human intelligence to help handle exceptions and novel situations. We believe this approach has potential for transforming the collective intelligence that is the large law firm.

## Introduction

A large law firm typically exhibits a collective intelligence comprised of hundreds or thousands of legal minds aimed at simultaneously engaging thousands of active matters across scores of industries and dozens of practice specialties with distinct doctrinal and procedural characteristics. The firm is challenged not only to achieve successful, cost-effective outcomes for its clients, but must also simultaneously, in competition with other firms and alternative service providers, attract and cultivate talent, develop and coordinate capabilities across multiple evolving areas of practice and continually improve a robust collective intelligence to gain a competitive edge. As various types of machine intelligences and tools are introduced, firms must also groom these into the collective. In this paper we explore a human-machine hybrid system for addressing this large scale, multi-dimensional, dynamic optimization challenge to coordinate the matching of incoming work to the collective intelligence of humans and machines. Machine intelligence is needed to handle the computational complexity and it is complemented by human intelligence to help handle exceptions and novel situations. We believe this approach has potential for transforming the collective intelligence that is the large law firm.

Traditionally, large law firms tend to deal with this challenge through massively distributed managers (often partners of the firm) overseeing clusters of lawyers segmented by geography, practice, industries, client teams, informal relationships, or even an *ad hoc* conglomeration of all of these. For illustrative purposes, instead of managing the collective intelligence of 1,000 lawyers as a global optimization problem, it would not be uncommon for a typical law firm to coordinate this as 40 or 50 separate groups of 5–25 lawyers. Managing lawyers would have visibility of incoming work and trade emails or discuss with other lawyers on a periodic basis to get a feel for their availability. Managing lawyers who have the time, interest, and emotional intelligence will get to know the talents and interests of their teams through lunches, working sessions, structured reviews, and “check-ins.” Machine intelligences are increasingly available in firms, but their use, even when directly relevant, can be subject to an assortment of factors including personal comfort with technology, ability to recognize applicability, real or perceived lack of time to learn new ways to do things, and even short-term economic considerations. Some firms put a lot of effort into knowledge management processes and systems to leverage the collective memory of the organization. These efforts can be extremely useful if they are maintained, and teams and individuals know how and where to find relevant information.

When these approaches work well, client needs are satisfied, and lawyers are closely mentored to hone their craft and business acumen. However, in this model, managers often don't have visibility of the entire pool of work assignments or of the talents and developmental needs of those who can work on them. Some lawyers may work 80-h weeks at the same time other lawyers in different clusters that could help are under-utilized. Lawyers could spend years gainfully employed on a single matter, but not develop the broader base of skills they will need to mature as a lawyer. Unless a more coordinated approach is taken and managers have augmented visibility of thousands of assignments and lawyers, staffing decisions would be significantly suboptimal for getting assignments done in an efficient manner and for building firm-wide capabilities for the long-term. Moreover, even if the fully informed visibility can be achieved, it would still be extraordinarily difficult for managers to handle the computational load of matching lawyers to assignments in a comprehensively considered fashion.

Coordinating and developing these intelligences at scale are challenge enough, but the task is further complicated by the increasingly dynamic staffing, workload, client-relations and financial demands. The Great Resignation, the COVID-19 pandemic, remote work arrangements and unprecedented competition for talent among firms are examples of tectonic shifts that present long-term implications.

Increasingly, machine and hybrid intelligences are becoming part of this networked collective intelligence with varying degrees of success. These include, but are not limited to, rule-based expert systems that can replicate certain legal processes, machine learning systems that can analyze documents for anomalies or clauses of concern, and workflow systems that help to coordinate legal processes across humans and machines (Armour and Sako, 2020; Waisberg and Hudek, 2021). Recently, large language models (Peters et al., 2017; Devlin et al., 2019) and generative artificial intelligences like ChatGPT are showing promise to support legal research, argument analysis, and legal document drafting (Ambrogi, 2023). While these additional intelligences can bring considerable capabilities to the table, they ironically also add to the coordination challenge and often must themselves be developed and adopted while being leveraged.

Synthesizing the diverse intelligences of a large law firm is a large-scale, multi-dimensional, dynamic optimization challenge which requires the scalability and computational power of machines as well as the situational awareness, social acuity, and practical flexibility of humans. One might expect that firms with high volumes of very similar matters might be further along toward optimizing the collective intelligence of their firms and may even be on their way to automating their work with forms and rule-based systems. Those with complex practices looking to solve difficult, cross geography, multi-practice challenges for lawyers will typically find this much more challenging. In this paper, we focus on one especially crucial nexus of law firm function–the selection, combination, and deployment of talent on client matters–to highlight the oddly anachronistic staffing method typically taken by even the most sophisticated law firms and to suggest an alternative approach that employs advances in computational methods and organizational design. We call this an “Intelligence Coordination System.” We draw conceptual inspiration from flexible information manufacturing paradigms to automatically construct intelligent systems (Kaomea and Page, 1997), as well as advances in methods for pattern matching, Pareto optimization and machine learning, toward achieving a “collective superintelligence” (Minsky, 1986; Bostrom, 2014; Yampolskiy, 2016; Malone, 2018) composed of many simpler intelligences.

**Figure 1:**
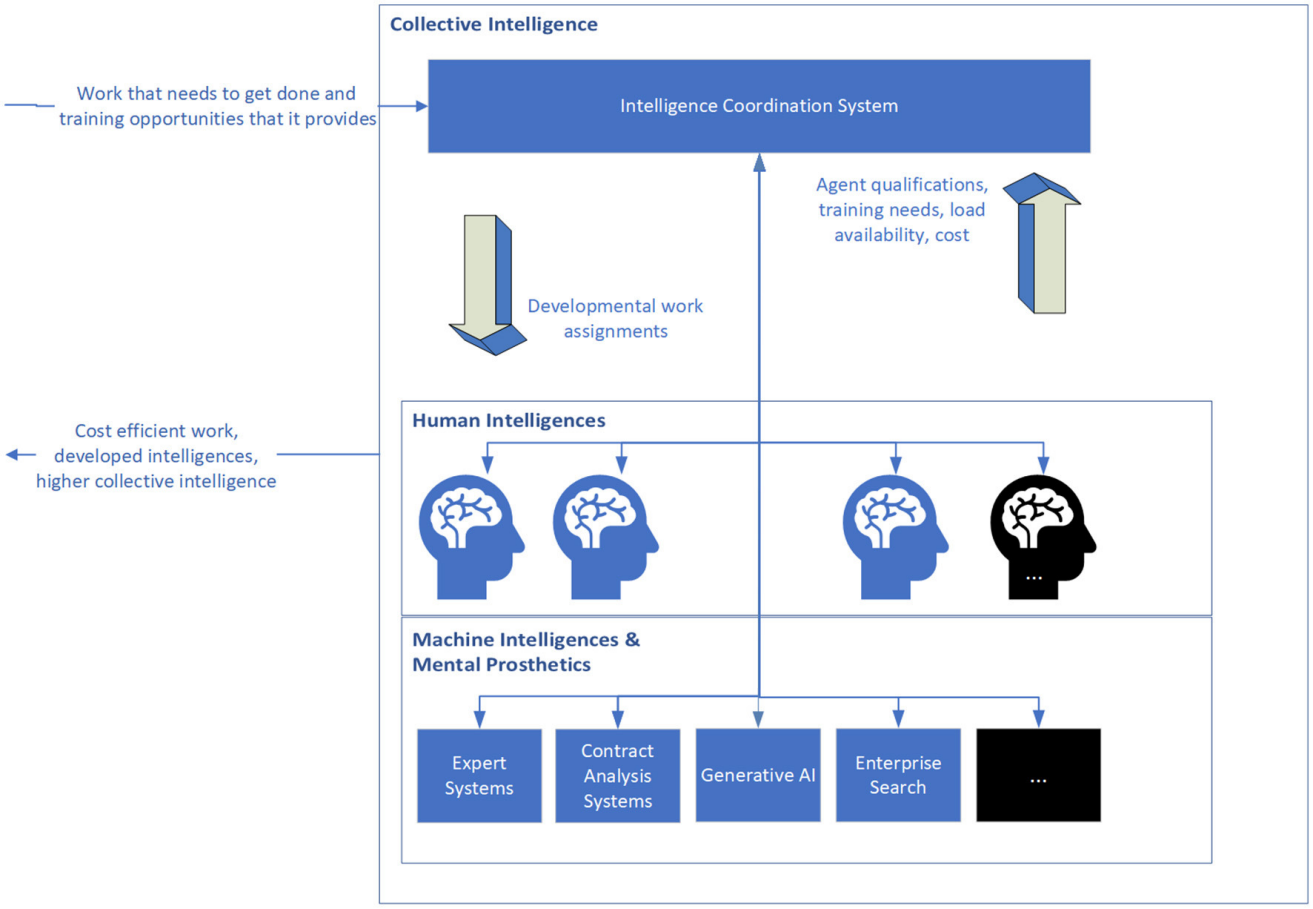
The intelligence coordination system coordinates work across human and machine intelligences to optimize the use and development of expertise, balance load and manage costs.

The Intelligence Coordination System (shown in Figure 1) acts as a sort of operating system for the law firm, recommending assignments which best leverage the particular qualifications of human and machine intelligences for work that needs to be done while also addressing the need to allocate assignments for the growth and development of these intelligences.

## System architecture

**Figure 2:**
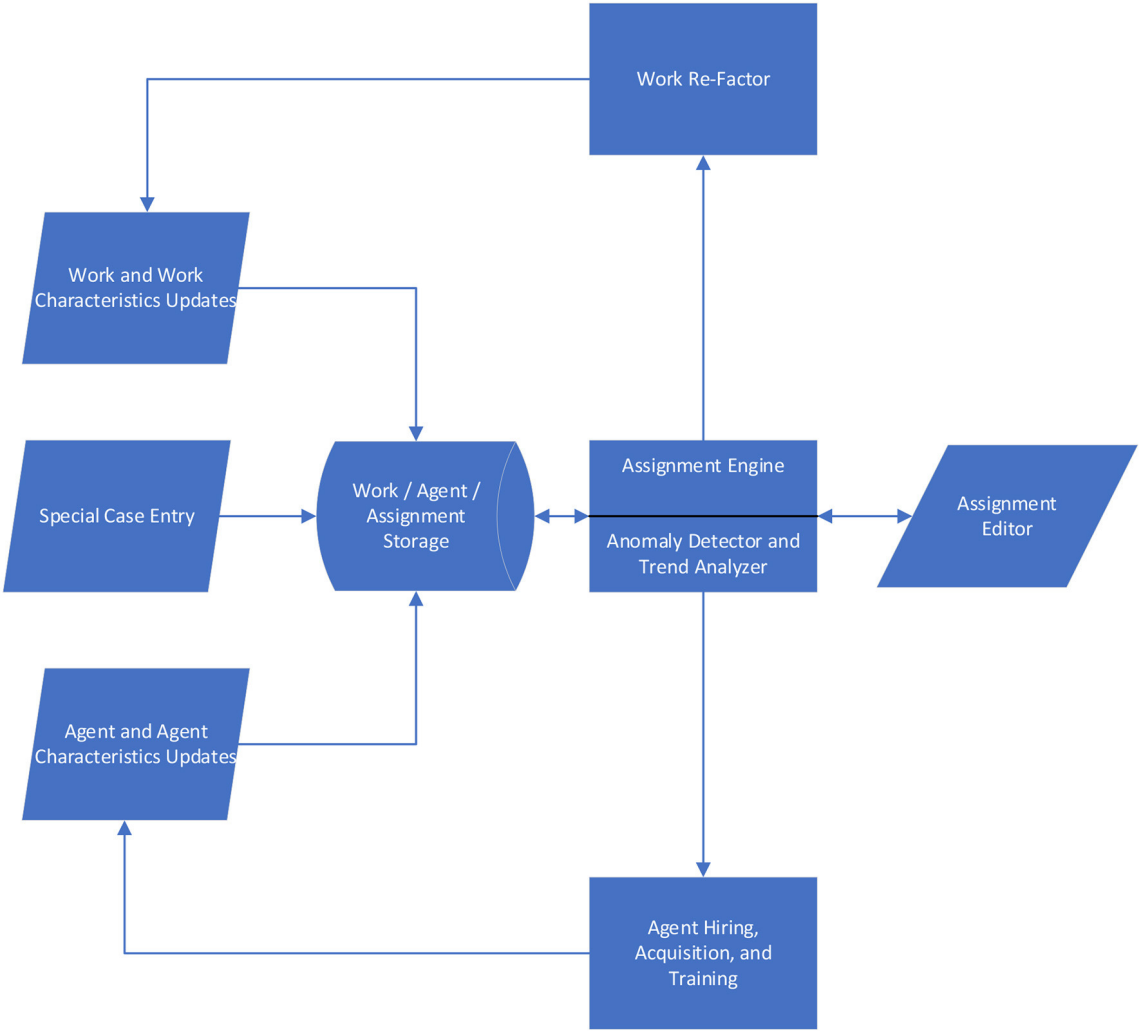
Architecture of intelligence coordination system.

Key components of the Intelligence Coordination System are shown in Figure 2. The core of the system is the Assignment Engine which attempts to optimally match incoming work that needs to be done with the most appropriate human and machine agents able to do the work. The Anomaly Detector and Trend Analyzer module helps to identify situations that may require systematic interventions to enable better staffing options–such as alternative knowledge management or workflow designs to improve performance on certain types of matters, or alternative recruitment and promotion strategies to address chronic staffing shortages in particular practice areas. The functions of both the Work Re-Factor module and the Agent Hiring, Acquisition, and Training module are currently envisioned to be human functions, although some parts of these functions could be automated in the future. The Assignment Editor also allows human modification of the assignments that the system proposes, to account for the range of nuances that ultimately should be accommodated but are not yet automatically addressed.

## Data structure and relationships

The Intelligence Coordination System takes as inputs information about work assignments (“Work” or “W”) to be undertaken as well as available human and machine agents (“Agents” or “A”) that can do Work. Managers will typically break client matters down into Work assignments that can be distributed amongst more junior lawyers. (While in this paper we will often focus on the human Agents who are lawyers, most of the concepts are readily applicable to paralegals, staff, and machine Agents.) It is important at this stage to consider comprehensively the client's needs to ensure an appropriate blend of legal expertise and other resources is allocated (see, e.g., Gardner, 2017; Gardner and Matviak, 2022). Similarly, it is important that human (and increasingly machine) resource departments structure and categorize the qualifications of Agents so that their skills can be optimally deployed. In this section we discuss how this information about Work and Agents is taken in and stored so that it can later be leveraged.

### Work

Work requires a set of qualifications (“Qualifications” or “Q”), represented by vector Q(W). Qualifications for a particular task might include authorization to practice law in a relevant jurisdiction, proficiency in a language likely to be used in the task, industry knowledge, experience with a particular client, experience drafting a particular type of brief or memorandum, knowledge of an area of law, successful completion of relevant training classes, positive references from particular managing lawyers, successfully completing similar work in the past and other relevant credentials or other indicia of competence or experience.

Work offers Training opportunities represented by vector T(W). Such training opportunities might include experience in preparing particular types of briefs, memoranda or other documents; leading particular kinds of assignments; or working with particular clients, or particular managing lawyers.

Work imposes a load or requirement of time (“Load” or “L”) represented by L(W). For purposes of this version of the model, we use a rough approximation of how long it will take to complete an assignment. It is expected that as the system is used, it would be possible to build more refined models of load estimations that vary depending on Agent skill level.

Work incurs a cost which clients are willing to pay (“Cost” or “C”) represented by C(W).

### Agents

An Agent has a set of Qualifications to do work represented by vector Q(A). The vector elements correspond to those of Q(W) so that we can easily check if an Agent is qualified for Work by stepping through each position of the vectors and seeing where Work-required Qualifications match with Agent available Qualifications.

An Agent may desire training opportunities (“Training” or “T”) represented by vector T(A). The vector elements correspond to those of T(W).

Agents have availability of time represented by scalar value L(A).

Agents have a Cost level represented by C(A).

We represent inputs to this version of the system as vectors for conciseness of notation as well as to inspire parallel computation in implementation. For purposes of this discussion, the values of the vectors are binary to simplify the description of the fundamental features of the concepts. We envision that some of the relevant parameters can be integer or real values in advanced implementations of the system to characterize both the circumstances and the choices in a more nuanced manner. Table 1 illustrates examples of Work and Agent Qualification and Training vectors.

**Table 1:** Simple example of qualification and training vectors for work and agents.

**Qualification**	**Work requires**	**Agent has**
Experience in writing briefs	Yes	Yes
Mid-level or above lawyer	Yes	Yes
Writes fluent French	No	Yes
Qualified to practice in New York	Yes	No
Qualified to practice in California	No	Yes
Strong client interaction skills	Yes	Yes
**Training**	**Work provides**	**Agent desires**
Experience in writing briefs	Yes	Yes
Courtroom experience	Yes	Yes
Experience in intellectual property litigation	Yes	Yes

The Qualification table illustrates an example in which a particular Agent meets the qualifications of being experienced in writing briefs and being a mid-level or above lawyer but is not qualified to practice in New York. In this example, the work meets all of the Agent's training desires.

Firms will typically need to find and evolve the balance of information that is most useful to collect vs. the technical and cultural changes required to collect it. Most firms will likely have classifications for the types of matters worked for clients, the amount of time each lawyer works on each matter, the training lawyers have received in Continuing Legal Education programs, the documents each lawyer writes or edits. Firms are increasingly asking lawyers to project their future availability to support the assignment process. AI systems are sometimes used to classify types of documents written and thus lawyer work on documents can reveal the types of experience they have had. Although many firms already spend resources to collect many of the types of data needed for this system, most will need to aggregate and groom the data into a standardized forms that can be used by the Intelligence Coordination System.

## Assignment engine operation

Perhaps the most important function of the Intelligence Coordination System is the Assignment Engine operation. It generates viable combinations of Agents and Work and finds Pareto optimal pairings across multiple objectives. To describe this function, we start by discussing how it constructs potential assignments. However, given the combinatorial growth of the assignment space, it must intelligently prune the space leveraging heuristic search methods (Chandra, 2020) before offering the pareto optimal sets for consideration.

### Constructing potential assignments

In order to construct potential assignments, the system considers how well Agents and Work align regarding Qualifications, Training, Load and Cost.

#### Qualifications for work

In order for Agent A to be fully qualified for a particular task, W, the qualifications required for that task would need to be a subset of the qualifications of that Agent: i.e., in vector notation, Q(W) · Q(A) = number of non-zero vector values of Q(W). It is possible that there is not an Agent fully qualified for a particular assignment in a given period. In that case, assuming for now that we consider all qualifications equally, MAX [Q(W) · Q(A)] over all W and A would be the most qualified Agent for the Work. These situations are discussed in the Unassigned Work section below.

#### Training for agents

For Agent A to achieve training objectives an assignment, W, T(A) intersect T(W) cannot be null. In vector notation, T(A) · T(W) ≠ 0. The best training opportunity W for Agent A in assignment period would be MAX [T(A) · T(W)] for all W.

#### Load

For an Agent to be assignable to Work, the Agent must have availability greater than or equal to the Load requirements of the Work: L(A) ≥ L(W). Whenever as assignment is made, L(A) is reduced by L(W). Artificially intelligent Agents might typically have higher L(A) values than human Agents since they can often work night and day without rest and scale up with additional hardware. Managing human Agent load helps to avoid burnout, improve retention, and enable longer-term team planning. Human L(A) in a given time period could be based on averaged work schedules, but adjusted for vacation periods, sickness, personal stamina or modified work schedules.

#### Cost

Lawyers typically come in cost bands. First year lawyers cost less than second year lawyers, who cost less than third year lawyers and so forth. Similarly, different classes of machine intelligences would have different Costs for use. We would typically want to assign work first to the lowest Cost option that satisfies a given set of requirements. We would want to avoid assignments where C(W) < C(A) and the Assignment Engine lays the foundation for cost projections to be systematically considered in real time at the point of initial staffing allocation.

### Managing the potential search space

Consider that there is an ordered list of N available work assignments and that for each kth assignment there are Mk fully qualified and Load-available, Agents to perform the work. Assuming each unit of work is assigned to only one Agent, there would be $\prod_{k=1}^{N}M_{k}$ combinations of assignment sets to consider.

In a simplified but illustrative example of a law firm with 1,000 lawyers that schedules work weekly, there might be 100 assignments and for each assignment there could be 10 fully qualified, available Agents. In this case there would be 10^100^ (or a googol set) to evaluate. Even with the computational power available today, it's not viable to take a brute force approach. At these magnitudes, the system needs to intelligently construct and search the space of potential assignment sets.

Assignment combinations are constrained by ethical walls. Lawyers are typically restricted from working on matters for which they may have a conflict of interest. A firm with hundreds of lawyers would usually have numerous such walls in place, and these will reduce the combinations of Work and Agents that need to be considered.

The search space can be reduced in the following ways with no loss of optimality:

- Where Work assignments have identical Qualification requirements, Training opportunities, Cost and Load requirements, we do not need to consider permutations of different orderings of these Work assignments.
- Where there are Agents which are identical in Work qualification, Training desires, Cost and Load, we need not consider permutations of different orderings of these Agents in creating assignments. This is more likely with junior lawyers and with machine Agents.

To the extent a firm decides that particular units of work can only be done by certain Agents and those Agents can only do those particular units of work, the search space can be segmented into smaller search spaces to “divide and conquer” the problem. Common dimensions which might result in such segmentation include but are not limited to the following:

- Work needs to be performed in a particular language, and certain Agents only work in that language.
- Work requires an Agent be legally qualified to practice in a particular jurisdiction, and some Agents may be only qualified to work in that jurisdiction.
- Work requires a particular area of expertise, and some Agents may be principally qualified to work in that area of expertise.
- A client may strongly prefer particular Agents to work on its matters.

It should be noted that while such segmentation is useful, one should be careful not to segment the space where not necessary. For example, certain types of Work might originate in a particular jurisdiction but may not be subject to jurisdictional restrictions that would call for this segmentation. Indeed, it may be beneficial for a particular assignment and for the firm at large to draw on a wider available pool of Agents.

Even with the reduction and segmentation of the search space, there will still be times when it is too large to be searched in the allotted time. In these cases, we would want to order the search such that it prioritizes Work, Cost, Load and Training in the order preferred by the firm for a particular assignment period or for a particular assignment type. Depending on preferences, the system would order the following operations:

- The system dynamically cycles through Work starting with the Work which has the fewest fully qualified, available Agents and progresses toward those for which there are the most. This helps to avoid “wasting” the time of highly specialized Agents with work that can be done by other Agents that don't have specialized expertise.
- Potential combinations of Work with fully qualified but lower-Cost Agents should be generated before those with higher Costs. For instance, clients will not want to pay for a senior lawyer to do work a more junior lawyer can do.
- Potential combinations of Work with fully qualified, but lower-Load Agents should be generated before those with higher Loads.
- Potential combinations where Training requirements are best fit should be considered before those where there is less of a fit.

In situations where there is not enough time to complete the search, a typical law firm might to prioritize Work, then Cost, then Load, then Training. Of course, the ordering can be altered for different prioritizations as desired.

These techniques enable a significantly more comprehensive and systematic approach for enumerating and evaluating staffing options, to match appropriately qualified Agents to Work, train Agents in areas they need to grow, to balance Load and to manage Costs than would typically be achieved in modern law firms through distributed and manual processes.

### Selecting an assignment set

An organization would typically want to make optimal choices balancing the need for getting Work done, minimizing Costs, balancing Load, and training Agents. As the system generates combinations of assignments consider that the following four scores are calculated for each set:

- Percent of Work assigned to fully qualified Agents;
- Cost efficiency of the assignments $=\overset{N}{\underset{k=1}{\sum }}\left(C\left(W_{k}\right)-C\left(A_{k}\right)\right)$ (For consistency of graphical representation, cost efficiency is represented as a percentage of the most efficient assignment set considered.);
- Percent of Agents with Load greater than lower expectation bound and less than upper expectation bound; and
- Percent of training objectives fulfilled.

**Figure 3:**
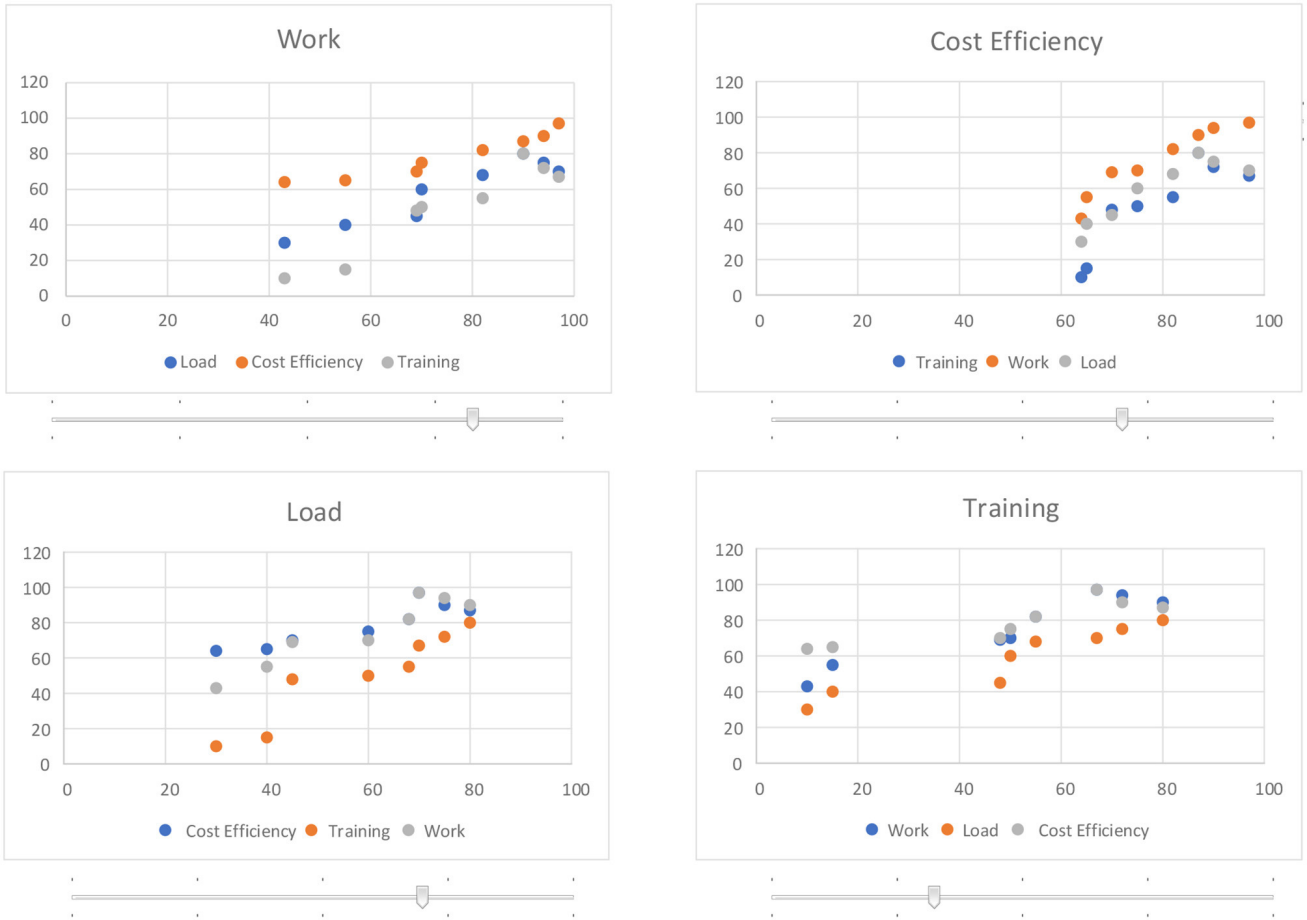
User interface allows dynamic navigation across four-dimensional objective space of assignment sets.

The user interface (shown in Figure 3) allows for navigation across the four-dimensional objective space of pareto efficient assignment sets. Moving the slider for one of the four objectives chooses the assignment sets which can attain that objective level. (It should be noted that the slider may not move smoothly, but instead could jump between levels since there may not be assignment sets that satisfy intermediate levels.) When one objective is set, all of the graphs are redrawn to reflect the remaining choices in the space.

### Special cases

Particular Work could be so critical or time sensitive that it should jump all the normal assignment rules. It may even require a specific, “hard wire” assignment of an Agent. Senior personnel in law firms are often familiar with special staffing accommodations to address matters that are in a “critical stage” or the strong preferences of a client or a senior lawyer. In extreme cases, Agents could be pulled from other assignments in light of such circumstances. While accommodation of such situations interferes with normal prioritizations, they help the system to adapt to exceptional needs. The ability of the system to quickly make good assignments and re-assignments in such situations is an additional point of value.

Analogously, there could be particular Agents who are prioritized to achieve particular training objectives. Perhaps they have unique talents but need to develop complementary skill sets to be able to realize the value of such talents on future Work assignments. These Agent assignments can be made ahead of the normal assignment process. To minimize Work impact, they might even be assigned to Work already staffed with another Agent.

### Anomaly detection and trend analysis

#### Unassigned work

**Figure 4:**
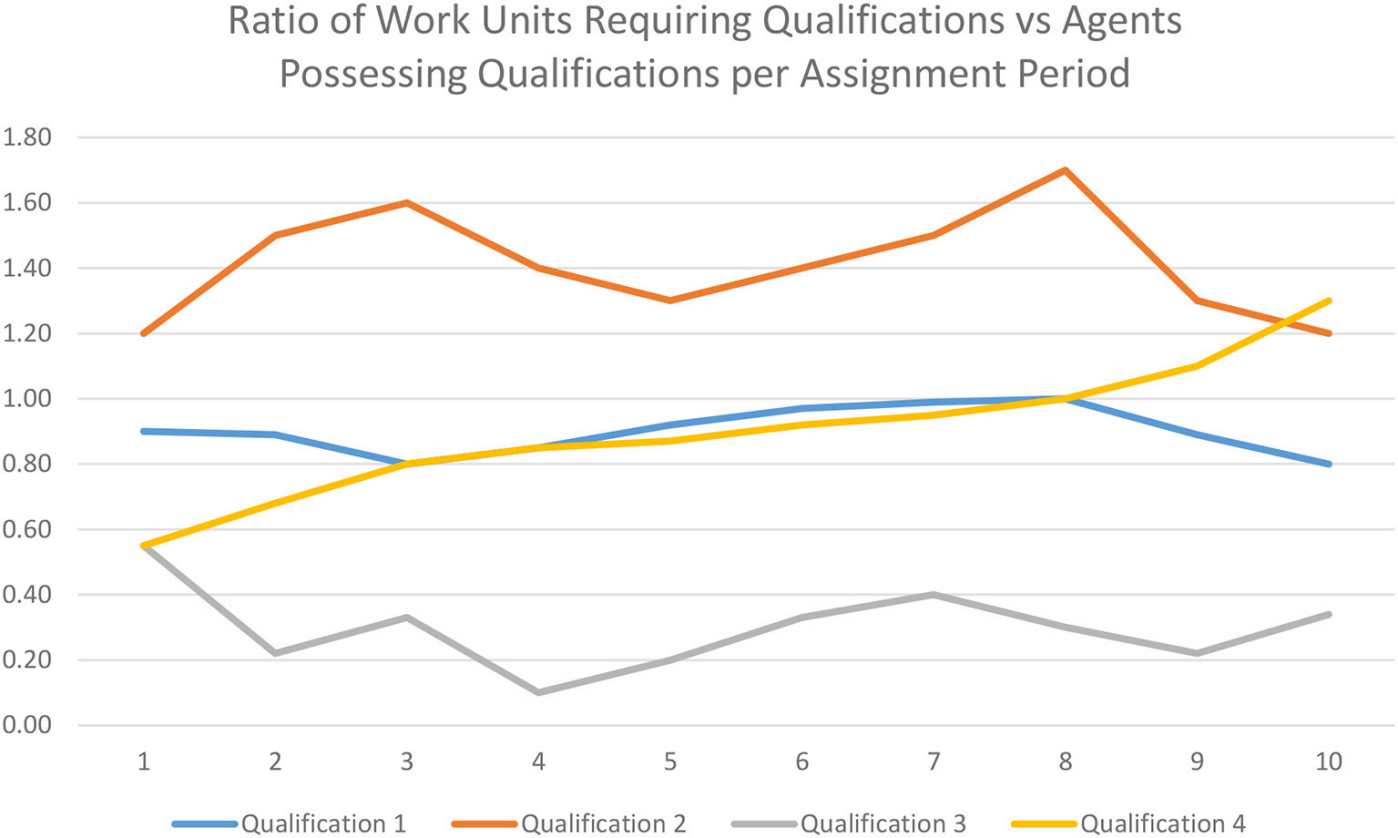
Sample graph of the Ratio of Work Units Requiring Qualifications vs. Agents Possessing Qualifications per Assignment Period. Qualification2 is chronically in short supply and likely needs to be addressed with the addition of new Agents or cross training of existing Agents. Qualification 3 is chronically in low demand and might imply less resources be dedicated to it.

It is possible that after running through the initial assignment process, there remains work that is unassigned. This can be addressed in a variety of ways:

- There may be fully qualified Agents who are assigned to other work and not able to take more Load under normal circumstances. In this case, all fully qualified Agents could be presented to a human operator ranked from the lowest to the highest Load and indicating the Training match. The human operator can choose whether to increase the Load of a particular qualified Agent with the otherwise unassigned Work. Depending on the circumstances, the human operator may instead choose to re-assign Work of the Agent to another. For this task, the Assignment Editor could show the human operator the rank order of the easiest to fulfill Work assignments and Agents qualified to fill them.
- There may be partially qualified Agents who have the Load capacity and can benefit significantly from the opportunity of engaging the unassigned Work. The human operator may choose to make such an assignment, realizing that additional supervision may be required. Such an assignment would typically need to be accompanied by an additional supervisory Work to coach the partially qualified Agent through the assignment.
- A particular type of Work may be chronically hard to assign because certain Qualifications are in short supply in the firm. (Figure 4 illustrates this with sample Qualification 2.) The Trend Analysis Module can identify the Qualifications most in demand for Work and least possessed by Agents. This might be addressable in the shorter run by re-factoring work or breaking it down into smaller pieces where more generic Qualifications can be fulfilled by a larger pool of Agents, leaving those Agents possessing the specialized Qualifications more Load capacity. Alternatively, the Training classes can be provided and assigned to less Loaded Agents to Qualify them for in demand Work or the organization can hire for the appropriate Qualifications.

#### Underutilized agents

There could be times when some Agents are persistently underutilized. This can happen with a sudden influx of new lawyers (such as the traditional summer and fall onboarding periods), with a slowdown of business in particular practice areas or geographies, or inability of a particular lawyer to meet firm standards. This can be handled in a variety of ways, depending on the situation.

- Agents can attend formal training. Training could be modeled as Work and would be entered with no requirements other than training pre-requisites. Banks of training should be in the ready for such periods. The system can help determine training that should be developed and ready based on types of work most done or types of work chronically short of Agents.
- Agents can be doubled onto Work in an unbilled Training status. Here, Load availability and Training fit are prioritized before Work Qualifications.
- Ultimately, the skillset or capabilities of a particular Agent may not be a fit and require special counseling.

#### Overutilized agents

There can also be times when Agents become overutilized. There can be busy M&A cycles for corporate lawyers, or a single new large litigation can consume large swaths of lawyer time. Industry phenomena like The Great Resignation of 2022 can draw down lawyer staffing levels at all levels of experience. Even more basic, it is recognized that when staffing decisions are made on long term matters, we cannot always predict the long-term loads of work and work demands for multiple practice areas can peak at similar times. An automated or semi-automated way to help a firm to adjust to these situations can help maintain production continuity for clients.

- When the system detects that an Agent is overloaded, there are a few paths it can take to resolve the situation. One approach is to re-assign some of the Agent's work to another. This is easier to do for work with lower Qualification requirements and when a matter is in a less critical stage.
- A more complex operation is to re-factor work so that others can help. For example, consider the task of authoring a merger agreement. An additional human can help by authoring particular sections that are fairly independent from other parts of the document. Systems can help by providing forms or agreements from similar deals to greatly reduce the amount of time it takes to complete the agreement.
- Moreover, chronic shortages in particular talent areas can be identified for action in recruitment, training, or other strategic measures.

## Integration of “ALIEN” machine intelligences into the collective intelligence

The formulation of our system to this point assumes that machine intelligences can be modeled and integrated similarly to human intelligences. While this is sometimes the case, (Martin, 2000) describes that “It is time to give up the twentieth-century notion that artificial intelligence is like human intelligence.” Some machine intelligences act as mental prosthetics. Modern day GPS systems in cars are a great example. With the help of satellites and route planning software, they keep a human driver moving efficiently toward their destination while the human performs the (at least so far not completely automated) task of manipulating the vehicle through busy streets teaming with people crossing against lights, erratic drivers, incorrect or obstructed road demarcations, etc. Other machine intelligences can replace humans at certain tasks altogether. (Waisberg and Hudek, 2021) discusses families of machine intelligences used in the legal industry. Here we discuss a few of them and how they can be integrated into our collective intelligence.

Contract Analysis Software is an application of artificial intelligence which typically acts as a sort of mental prosthetic. It that can significantly reduce the Load it takes to perform contract diligence review. Introduction of these Agents into an organization typically decreases the human Load for diligence Work, increases the need for Qualifications of human Agents to be trained to use the software and creates Work for human Agents to review or supervise work done by the machine Agents.

Expert system intelligences automate the consideration of legal questions which can be codified into arbitrarily complex sets of if–then rules. These systems do require Work to codify, but once they are created, can provide “…efficient, scalable, answers to relatively routine legal questions at high volume and low cost.” Virtually all the Work codified in this way could be handled by these expert system Agents, freeing up nearly all of the human Agent time that would otherwise be dedicated to this Work.

Enterprise search systems provide a means for Agents to find historical work product, methods, processes, precedents, etc. similar to a new unit of Work that must be completed. Think of the power that Google provides to everyday life. Enterprise search systems index all relevant information of an organization and helps to serve as a memory for the collective mind. As human Agents come and go, their work product can remain available to the collective intelligence so that it is leverageable for future work. While such search systems are not always considered “artificial intelligence,” search is at the heart of many AI systems and can certainly play a major role for a collective intelligence. A well-constructed and maintained enterprise search system can significantly reduce the Load for researching and drafting documents or for bringing new Agents up to speed on a deal that is re-starting or a litigation that has been going on for years. In doing so it can also reduce errors and increase the quality of Work.

## Summary and areas for future work

We have presented here a theoretical system for managing Work allocation amongst Agents for large law firms in a way that attempts to optimize leveraging of key Qualifications, balances Load, manages Costs and Trains Agents. Cracking this challenge can help improve lawyer retention by managing the highs and lows of Load to avoid burnout and disengagement. Ensuring a balance of Training helps lawyers to grow in their craft and become more productive over time. Driving work to lower cost Agents (whether they be human or artificial intelligences) is more efficient for clients and helps firms to improve their ratio of senior to junior lawyers. All of this helps law firms to be more profitable, clients to get a more efficient process and lawyers to grow in their careers.

Many of the technical capabilities to make such a system are available, but as is often the case in AI, capturing the data to make it work in a suitable format, getting it to perform in a complex real-world setting and ensuring it fails in a graceful manner are real challenges that remain. It is expected that a law firm would focus initially on collecting and grooming the information which will most significantly impact its bottom line. Assuming a law firm of 1,000 lawyers charging an average of $750 per h, improving utilization for each lawyer by a net of just 10 min per day would generate $125 thousand per day. In a firm constrained by a lack of work, this valuable time could be spent training. It can easily cost hundreds of thousands of dollars to recruit, incent even a junior lawyer to join a firm not to mention train them to be productive. Avoiding or delaying the regretted loss of a productive lawyer by complementing personal mentoring with systematic alignment of work and training to interests and availability can produce tremendous economic value for a firm.

While the heart of the optimization system as described is primarily structured as a heuristic search optimization problem, machine learning is likely to be employed in various aspects of information collection and potentially in the trend analysis and exception handling stages as system use generates sufficient amounts of data to make it possible.

The system is expected to be tested in two vastly different settings. First, in a large, diverse law firm and additionally in work allocation for a drone swarm.

## Data availability statement

The original contributions presented in the study are included in the article/supplementary material, further inquiries can be directed to the corresponding author.

## Author contributions

The author confirms being the sole contributor of this work and has approved it for publication.
